# Neuroactive hormones and personal growth: associations in Chilean adolescents (ages 12–25) with ovulatory dysfunction

**DOI:** 10.3389/fpsyg.2024.1433437

**Published:** 2024-08-21

**Authors:** Juan Pablo del Río, Valeska Tapia, Hugo Soto, Pilar Vigil

**Affiliations:** ^1^Departamento de Psiquiatría y Salud Mental de la Infancia y de la Adolescencia, Facultad de Medicina, Universidad de Chile, Santiago, Chile; ^2^Millennium Nucleus to Improve the Mental Health of Adolescents and Youths Imhay, Santiago, Chile; ^3^Reproductive Health Research Institute, Santiago, Chile

**Keywords:** personal growth, neurosteroids, ovulatory dysfunction, adolescents, developmental psychology

## Abstract

**Introduction:**

Hormones produced by the hypothalamic–pituitary–adrenal-gonadal (HPAG) axis are crucial for modulating central nervous system (CNS) function and development throughout a person’s life. Disruptions in HPAG function can impact psychological development, particularly during adolescence—a period marked by psychological growth and the maturation of the HPAG axis. An early indicator of HPAG alterations is ovulatory dysfunction (OD), a common condition among adolescents.

**Methods:**

This study explored the associations between neuroactive hormones and personal growth in adolescents with OD. Female participants aged 12–25 years with OD were recruited, and assessments were conducted to profile their basic hormonal levels and various dimensions of individual development, including self-concept clarity, sense of coherence, self-esteem, perfectionism, self-control, and mood states.

**Results:**

Adolescents with OD (*n* = 117) had lower self-concept clarity and self-esteem compared to reference data. A significant portion of the sample displayed elevated levels of tension (71.25%), confusion (62.5%), fatigue (58.22%), and depression (52.6%). Self-esteem scores were negatively correlated with DHEAS (*r* = −0.224; *p* = 0.026) and glucose (*r* = −0.249; *p* = 0.010). Higher levels of free testosterone were associated with increased depression scores (coef = 0.2398; *p* = 0.002), whereas higher estradiol levels were linked to lower aggressiveness scores (coef = −0.0648; *p* = 0.001).

**Discussion:**

These findings indicate that hormonal imbalances in adolescents with OD could affect personal growth. Further research is needed to establish causal relationships between the variables considered.

## 1 Introduction

Personal growth can be conceptualized as a progression toward authenticity and life satisfaction (Rogers, 1959), integrating various individual aspects (Luis et al., 2022) and encompassing coexistence with others (Orón, 2015; Guzmán-Montiel, 2019). To thoroughly assess personal growth, it is important to consider psychological constructs such as self-awareness, openness to new experiences and change, existential persistence, independence, self-responsibility, self-compassion, and empathy toward others (Maurer et al., 2023).

Although personal growth is a continuous process, adolescence is a crucial stage (Erikson, 1968). This period encompasses psychological development and the maturation of significant physiological systems, including the hypothalamic–pituitary–adrenal-gonadal (HPAG) axis (Spaziani et al., 2021). The hormones produced by the HPAG axis are pivotal in regulating brain function and maturation throughout life, demonstrating the alignment between psychological and biological development (Merleau-Ponty, 2002). In fact, hormones, such as androgens or estrogens, can be synthesized outside the central nervous system (CNS), cross the blood–brain barrier, and regulate neuronal activity (Zheng, 2009).

Neuroactive hormones, such as steroids and peptides, play a critical role in brain maturation (Zweifel and O’Brien, 1997; Vigil et al., 2011; Mueller et al., 2014; Schulz and Sisk, 2016) and influence neurotransmission processes. Their effects on the CNS are classified as organizational (permanent structural changes) or activational (modulation of neuronal activity) (Arnold, 1985). Organizational effects include myelination, apoptosis, dendritic spine remodeling, and neuronal pruning, while activational effects can rapidly impact neurotransmitter systems (Schiller et al., 2016; Krolick et al., 2018). These activities are partly due to the widespread expression and activation of steroid hormone receptors and p450 aromatase in CNS cells, including neurons, astrocytes, microglia, oligodendrocytes, and Schwann cells (Montelli et al., 2012; Azcoitia et al., 2021).

The hormonal influence on the CNS is particularly significant for women. Fluctuations in sex hormones throughout a woman’s life significantly impact susceptibility to mood disorders, such as premenstrual dysphoric disorder, postpartum depression, and perimenopausal depression (Bloch et al., 2003; Smith et al., 2014; Sundström-Poromaa and Gingnell, 2014). Hormones from the HPAG axis also affect cognitive functions such as decision-making, emotional recognition, and memory (Hampson, 1990; Sundström-Poromaa and Gingnell, 2014; Pillerová et al., 2022). Moreover, disruptions in the HPAG axis can negatively affect mental wellbeing and identity development (Vigil et al., 2016). For example, polycystic ovary syndrome (PCOS), the most common endocrine disorder in reproductive-age women, is linked to a high prevalence of psychological symptoms like melancholy, sadness, and depression, worsened by menstrual irregularities (Shakerardekani et al., 2011).

Ovulatory dysfunction (OD) is an early sign of HPAG disruptions, with abnormalities in ovulation and irregular cycles often being the first indicators of underlying endocrinological alterations (Vigil et al., 2017). Since the hormones that regulate ovulation also influence the CNS, OD could potentially affect CNS maturation and psychological development (Rahiminejad et al., 2014; Sundström-Poromaa and Gingnell, 2014). However, few studies have specifically examined the associations between OD and psychological development. We hypothesize that OD in adolescents is associated with alterations in psychological aspects of personal growth, mediated by key hormones involved in neural and HPAG axis functioning. Therefore, the study objective is to characterize the possible association between psychological aspects of personal growth and key hormones involved in neural and HPAG axis functioning in a cohort of Chilean adolescents with OD.

## 2 Methods

### 2.1 Sample

A cross-sectional study was conducted. Chilean female Caucasian adolescents aged 12–25 years with diagnosed OD from a private practice center were recruited. All participants were at least 2 years post-menarche. Information was collected on the reason for consultation, educational background, current medications, and drug/alcohol use. According to the patient’s condition, OD was diagnosed based on established criteria (Vigil et al., 2017): two or more consecutive short or long menstrual cycles (<24 or > 36 days) or three or more such cycles within a year; a persistent luteal phase shorter than 9 days (determined through mucus symptom charting); serum progesterone levels below 5 ng/mL on day 21; or evidence of anovulation by ultrasound. Exclusion criteria included: patients with no OD diagnosis, lack of necessary information for minimum characterization, history of psychiatric hospitalization, major psychiatric diagnosis, hormonal contraceptive use, insulin-sensitizers use, and corticosteroid use. Convenience sampling was employed due to the study’s non-randomized nature. The Reproductive Health Research Institute (RHRI) ethics committee approved the study protocol. Informed consent for data analysis was obtained from all participants.

### 2.2 Clinical evaluation

Each patient underwent a complete clinical evaluation, and a serum basic hormonal profile (BHP) was conducted. The BHP involved testing for FSH, estrogen, thyroid stimulating hormone (TSH), T4 concentration, prolactin, dehydroepiandrosterone sulfate (DHEAS), androstenedione, total testosterone, calculated free testosterone [with albumin and sex hormone binding globulin (SHBG)] and 17-OH-Progesterone. A five-point oral glucose tolerance test with insulin (OGTT-I) was also performed. For menstruating patients, blood samples were collected on days 3, 4, or 5 of the menstrual cycle. For patients with amenorrhea, the BHP was conducted on any convenient day. All samples were collected in a fasting state before 9:00 am. The hormonal evaluation and diagnostic assessment of underlying endocrinopathies were performed as follows (Table 1).

**Table 1:** Descriptive data for the basic hormonal profile.

Hormone	Unit	P50	IQR	Method
FSH	mIU/mL	5.2	2.77	ECLIA
Estrogen	pg/mL	46.4	47.2	ECLIA
Total testosterone	ng/dL	37.8	26.2	ECLIA
Free testosterone	pg/mL	4.28	4.75	Calculated
SHBG	nmol/L	61.25	40.45	ECLIA
Androstenedione	nmol/L	2.5	1.25	ECLIA
DHEAS	ug/dL	213.5	143	ECLIA
Prolactin	ng/mL	10.5	7	ECLIA
TSH	mIU/L	1.89	1.31	ECLIA
HOMA	–	1.84	1.47	Calculated
I0*G60	–	958.5	845.7	Calculated

Unit, median and interquartile range and method are presented.

### 2.3 Insulin and glucose assays

The OGTT-I assay was performed following a 12-h fast. Participants received 75 g of glucose, and blood samples were obtained at 0, 30, 60, 90, and 120 min to measure glucose and insulin levels. Insulin resistance was diagnosed based on the presence of any of the following criteria: homeostatic model assessment (HOMA) index >2.09 (Burrows et al., 2015) insulin sensitivity index (ISI) composite <4.45 (Matsuda and DeFronzo, 1999; Takahara et al., 2013) or I0*G60 > 1,110 (basal insulin* glucose at 60 min) (Contreras et al., 2019).

### 2.4 Hyperandrogenemia

Hyperandrogenemia was diagnosed if any of the following criteria were met: total testosterone >47 ng/dL, free testosterone >9 pg./mL, DHEAS >250 μg/dL (Chen et al., 2021; Strauss et al., 2024), or androstenedione >2.8 ng/mL (Vigil et al., 2007). Total testosterone levels were measured using an enzyme immunoassay, and SHBG and DHEAS levels were measured using an immunoradiometric assay. Free testosterone levels were calculated using established methods that include total testosterone, SHBG, and albumin values (Vermeulen et al., 1999). 17-OH-Progesterone was also measured to rule out congenital adrenal hyperplasia (Hughes, 2009).

### 2.5 Hyperprolactinemia

Hyperprolactinemia was diagnosed if basal prolactin levels were > 25 ng/mL (Majumdar and Mangal, 2013). Low macro prolactin reactivity reading methods were used to rule out this condition (Vaishya et al., 2010). Prolactin levels were measured using immunoradiometric assays.

### 2.6 Thyroid disorders

Thyroid hormones were determined using Direct Quimioluminiscence. Primary hypothyroidism was diagnosed if TSH values were > 10 μIU/mL. Subclinical hypothyroidism (SCH) was diagnosed when basal TSH level was >5 μIU/mL and T4 values were within a normal range (Dhillon-Smith et al., 2020).

### 2.7 Characterization of personal growth

At the time of the first hormonal measurement and within the first 30 days following OD diagnosis, participants went through a personal growth assessment. This evaluation was designed to account for both self-development aspects and consider interpersonal relationships. It included the following instruments:

Profile of mood states (POMS) (Norcross et al., 1984): a 65-item scale measuring 6 different dimensions of mood states during the last 2 weeks (scores vary by subscale). It includes tension or anxiety, anger or hostility, vigor, fatigue or inertia, depression, and confusion.

Self-concept clarity scale (SCC) (Campbell et al., 1996): a 12-item Likert scale measuring self-concept clarity (scores range from 12 to 60). Self-Concept Clarity references a structural aspect of the self-concept: the extent to which self-beliefs are clearly and confidently defined, internally consistent, and stable.

Sense of coherence questionnaire (SOC-13) (Antonovsky, 1993): a 13-item Likert scale assessing sense of coherence (scores range from 13 to 91). Sense of coherence describes how an individual perceives the world as comprehensible, manageable, and meaningful.

Rosenberg self-esteem scale (RSES) (Rosenberg, 1965): a 10-item scale measuring self-esteem (scores range from 0 to 30). It is defined as one’s overall sense of worthiness as a person.

Frost multidimensional perfectionism scale (FMPS) (Frost et al., 1990): a 35-item Likert scale assessing different dimensions of perfectionism (scores vary by subscale). It encompasses concern over mistakes, doubts about actions, personal standards, parental expectations, parental criticism, and organization.

Self-control scale (SCS) (Tangney et al., 2004): a 36-item scale measuring self-control across various domains (scores vary by subscale). It can be defined as the capacity to change and adapt the self to produce a better, more optimal fit between the self and the world. Central to this concept is the ability to override one’s inner and immediate response, as well as to interrupt undesired behavioral tendencies and refrain from acting on them.

### 2.8 Statistical analysis

The Shapiro–Wilk test was employed to assess the normality of each dataset. Descriptive statistics were reported as mean [standard deviation (SD)] for parametric data or median [interquartile range (IQR)] for non-parametric data. Categorical variables were presented as absolute counts and percentages (%). A *t*-test was used to compare continuous data, while the Mann–Whitney’s *U-*test was utilized for non-parametric data. Fisher’s exact test was employed for the comparison of categorical datasets. ANOVA and ANCOVA methods were used for variance analysis. For multivariate analysis, multiple linear and logistic regressions were considered. Pearson or Spearman correlation indices were regarded as appropriate. A *p*-value <0.05 was considered statistically significant. A power analysis was not conducted due to the exploratory nature of this study. Statistical analyses were conducted using Stata v12.0 (StataCorp, TX, United States).

## 3 Results

The initial sample consisted of a total of 121 patients who completed the evaluation. After applying the exclusion criteria, the final sample comprised 117 patients with a median age of 18 years (IQR: 6 years). Mean BMI was 22.97 (IQR: 4.86). Regarding BMI classification, 59.4% were categorized as normal weight, 31.3% overweight, 6.3% underweight, and 3.1% obese. Menstrual Irregularity was the most common presenting complaint (51.3%). Other reported symptoms included acne (43.8%), weight gain (37.5%), and dysmenorrhea (18.8%). Regarding diagnosis, insulin resistance (55.5%) (IR) and hyperandrogenemia (54.8%) were the most frequent diagnoses. Additionally, subclinical hypothyroidism (29.6%), hyperprolactinemia (10.5%), (7.0%), and primary hypothyroidism (2.5%) were documented. These findings align with the most frequent causes of ovulatory dysfunction (OD) (Munro et al., 2022). Descriptive data for the BHP results can be consulted in Table 1.

**Figure 1:**
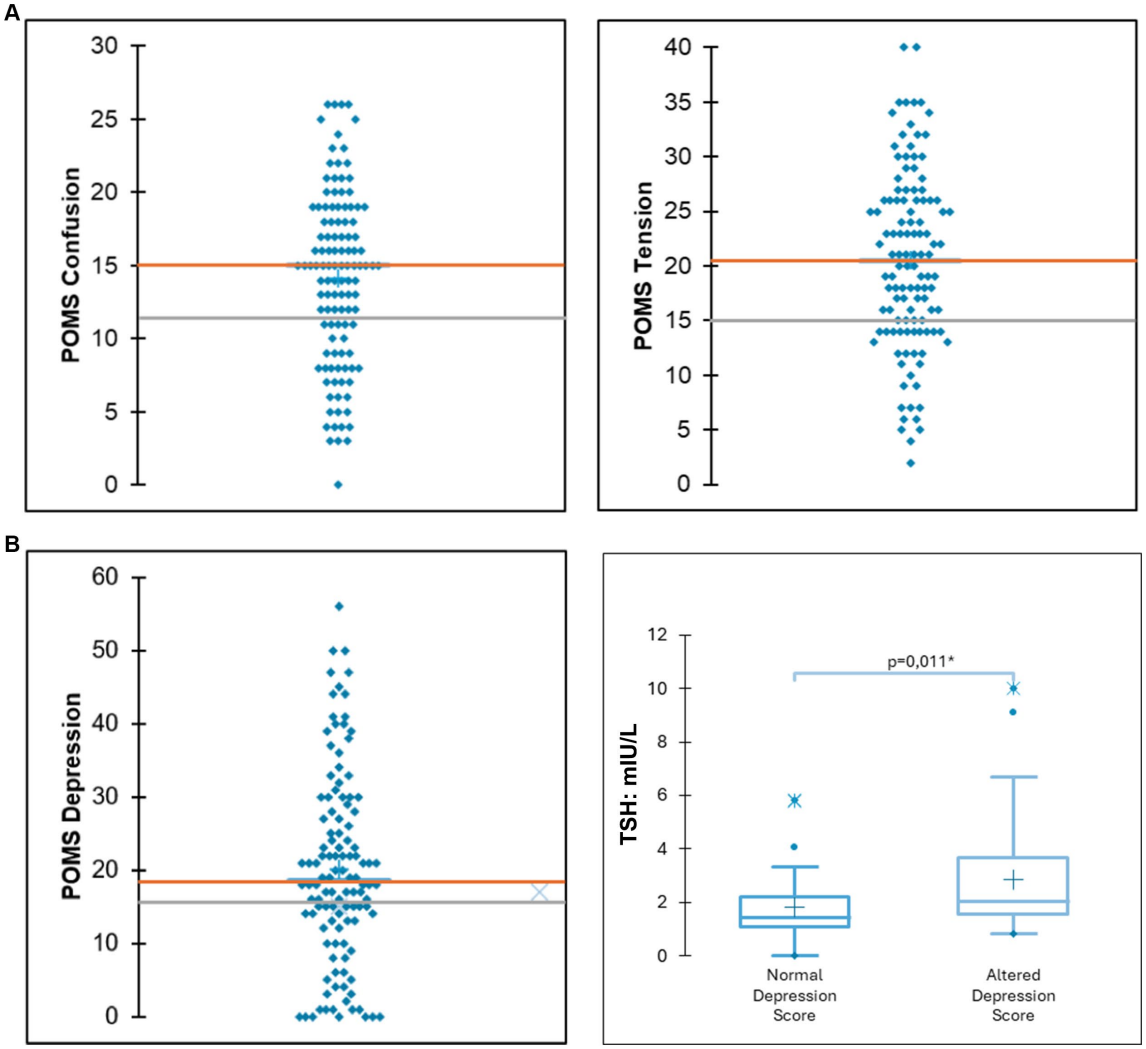
**(A)** Distribution of scores for depression and tension. The orange line indicates the mean scores of the study participants, while the grey line denotes the mean scores from reference data for the general population. **(B)** Among participants exhibiting elevated depression scores, significantly higher thyroid-stimulating hormone (TSH) levels were observed.

According to the POMS questionnaire, a significant portion of the sample exhibited elevated scores for tension (71.25%), confusion (62.5%), fatigue (58.22%), depression (52.6%), and aggressiveness (33.75%). Among those participants who exhibited altered scores for depression, a significantly higher TSH value was observed (5.23 mIU/L vs. 1.81 mIU/L; *p* = 0.001) (Figure 1). Compared to reference data, adolescents with OD displayed lower self-concept clarity and self-esteem scores (Gutierrez, 2015). The summary of other considered scales (SCC, SOC-13, RSES, FMPS, SCS), next to reference data, can be found in Table 2.

**Table 2:** Summary of scores in the studied population vs. normative reference for age range.

Scale	Evaluated dimention	Mean (X), standard deviation (SD) (reference sample)	Mean (X), standard deviation (SD) (study sample) *N* = 117; 12–25 years old	References
Self-concept clarity scale (SCC)	Structural aspect of the self-concept: the extent to which self-beliefs are clearly and confidently defined, internally consistent and stable.	12–18 years old (X = 34.68, SD = 7.05)	X = 40.3, SD = 10.3	Darcourt (2015) and Gutierrez (2015)
		18–34 years old (X = 37.07, SD = 9.58)		
Sense of coherence questionnaire (SOC-13)	Describes how an individual perceives the world as comprehensible, manageable, and meaningful.	X = 53.6, SD = 11.2	X = 53.6, SD = 5.8	Álvarez Rodríguez (2022)
Rosenberg self-esteem scale (RSES)	One’s overall sense of worthiness as a person.	18–25 years old (X = 19.67, SD = 6.63)	X = 19.9, SD = 5.2	Sinclair et al. (2010)
		26–35 years old (X = 22.28, SD = 5.66)		
Frost multidimensional perfectionism scale (FMPS)	It encompasses concern over mistakes, doubts about actions, personal standards, parental expectations, parental criticism, and organization.	Parental criticism (X = 7.3, SD = 3.5).	Parental criticism (X = 8.23, SD = 3.91)	Mandiola et al. (2022)
		Parental expectations (X = 13.6, SD =4.7)	Parental expectations (X = 12.68, SD 4.49)	
		Parental standards (X = 23.4, SD =5.2)	Parental standards (X = 22.36, SD = 5.68)	
		Concern over mistakes (X = 22.4, SD = 7.3)	Concern over mistakes (X = 20.5, SD = 8.28)	
		Doubts about actions (X = 12.4, SD = 4.3).	Doubts about actions (X = 11.63, SD = 3.90)	
		Organization (X = 23.6, SD = 4.2).	Organization (X = 23.06, SD = 5.56)	
Self-control scale (SCS)	The capacity to change and adapt the self to produce a better, more optimal fit between the self and the world. Central to this concept is the ability to override one’s inner and immediate response, as well as to interrupt undesired behavioral tendencies and refrain from acting on them.	X = 114.47, SD =18.81	X = 117.41, SD = 21.52	Tangney et al. (2018)

The following domains were considered: Self-concept clarity, sense of coherence, self-esteem, perfectionism and self-control.

**Figure 2:**
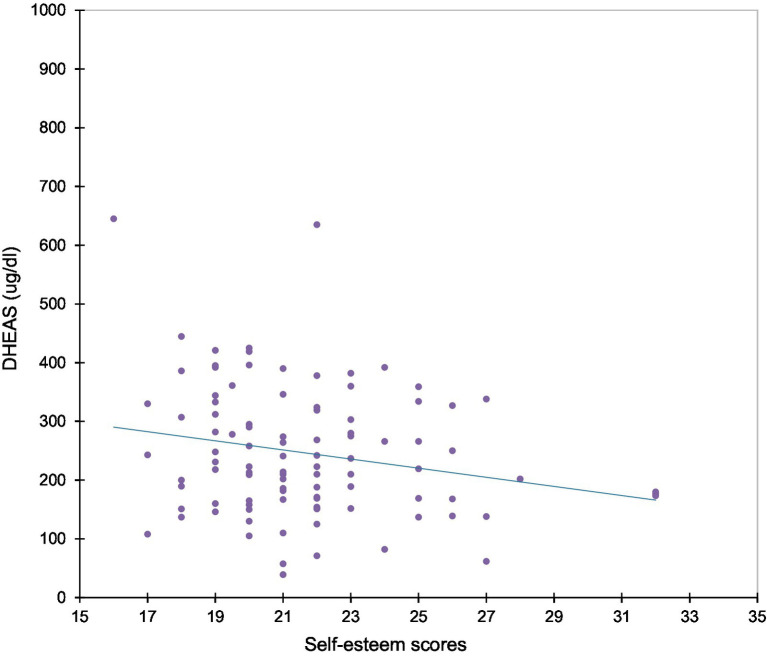
Correlation between dehydroepiandrosterone sulfate (DHEAS) levels and self-esteem scores. Higher levels of DHEAS were associated with lower self-esteem scores (*P* = 0.011; *R* = −0.224).

When multivariate analysis was considered, logistic regressions revealed positive correlations between the complaint of acne and scores for concern about mistakes (*r* = 0.217, *p* = 0.009), doubts about actions (*r* = 0.251, *p* = 0.007), tension (*r* = 0.296, *p* = 0.02), confusion (*r* = 0.279, *p* = 0.003), depression (*r* = 0.343, *p* = 0.001), and anger (*r* = 0.302, *p* = 0.001). Metrorrhagia was positively correlated with the level of fatigue (*r* = 0.225; *p* = 0.17). Weight gain complaints were negatively correlated with self-esteem (*r* = −0.231, *p* = 0.013) and personal standards (*r* = −0.255, *p* = 0.008). Considering hormonal measurements, self-esteem showed negative correlations with DHEAS (*r* = − 0.224; *p* = 0.026), insulin (*r* = −0.191; *p* = 0.049), and glucose (*r* = −0.249; *p* = 0.010) levels, even after adjusting for BMI (Figure 2). Fatigue scores were positively correlated with prolactin (*r* = 0.232; *p* = 0.20). When variance analysis was considered, it was seen that the score for tension increased with higher levels of TSH (coef = 1.198; *p* = 0.045) and free testosterone (coef = 194.5; *p* = 0.011). Depression scores increased with higher levels of SHBG (coef = 0.2398; *p* = 0.002) and similarly with DHEA-S (coef = 0.0867; *p* = 0.011). Aggressiveness scores showed an increase with higher serum levels of TSH (coef = 1.818; *p* = 0.004), DHEA-S (coef = 0.0515; *p* = 0.001), Free Testosterone (coef = 269.1; *p* = 0.001), and SHBG (coef = 0.1231; *p* = 0.001). Conversely, aggressiveness scores decrease with higher levels of estradiol (coef = −0.0648; *p* = 0.001).

## 4 Discussion

This study investigated the associations between neuroactive hormone levels, personal growth domains, and mood states in adolescents with ovulatory dysfunction (OD). Mood assessment revealed a high prevalence of tension, confusion, fatigue, depression, and aggressiveness within the study population (Shahid et al., 2011). Typical symptoms of OD, such as acne and weight gain, were correlated with scores for concern about mistakes, doubts about actions, tension, confusion, lower self-esteem, and personal standards. These results suggest that adolescents with OD may present a profile of personal growth dimensions different from that of the general population, which may be associated with hormonal dysfunctions.

Hyperandrogenism was a common diagnosis in this population (54.8%). Serum levels of androgens, like DHEAS and free testosterone, negatively correlated with self-esteem and positively correlated with depression, tension, and aggression. This aligns with the established role of androgens in modulating CNS function throughout life, particularly during adolescence (Laube et al., 2020). This action has functional and behavioral implications. Studies have shown that the normal increase in testosterone during 2 years of pubertal maturation was associated with increased activity in the amygdala in boys and girls. This was associated with increased levels of withdrawal temperament, consistent with an increase in threat sensitivity (Peters et al., 2015). Furthermore, a recent study shows that testosterone correlates with lower resting state functional connectivity between the amygdala and right superior frontal gyrus, but only in women (Kogler et al., 2023).

Up to 55% of participants exhibited IR, a condition usually associated with weight gain (Vigil et al., 2007). Weight gain was correlated with lower self-esteem and personal standards in our population. Notably, multivariate analysis showed that insulin and glucose were associated with lower self-esteem scores, even after adjusting for BMI. Existing research supports this link, demonstrating an increased risk of depression in individuals with IR, which persists when adjusted for BMI or age (Santoro et al., 1999). This would be consistent with animal models, where inactivation of the insulin receptor in the hypothalamus has been shown to result in systemic IR, dyslipidemia, and depressive-like behavior (Randolph Jr et al., 2011). Additionally, studies using functional magnetic resonance imaging in patients with PCOS and IR revealed alterations in limbic system activation during emotional tasks, which normalized after metformin treatment (Pike et al., 2009).

Other common diagnoses were subclinical hypothyroidism, primary hypothyroidism, and hyperprolactinemia. Our results show that patient with altered scores for depression exhibited higher TSH levels. This is coherent with the well-established relationship between thyroid disorders and mood alterations (Bauer et al., 2003, 2008; Singh and Sundaresh, 2022). Regarding hyperprolactinemia, our results show a positive correlation between prolactin levels and fatigue scores. Several studies have indicated that patients with hyperprolactinemia are more likely to experience depression, anxiety, and hostility (Fava et al., 1983; Liao and Bai, 2014; An et al., 2016). Even though there is still a lack of understanding of the mechanism that rules this relationship, prolactin can act on different brain regions, including those involved in neural growth, development, sleep, learning, and memory (Cabrera-Reyes et al., 2017).

It is also important to acknowledge the potential impact of body image concerns, often triggered by symptoms like hirsutism and acne, on self-esteem and emotional wellbeing (Whitlock et al., 2006). Importantly, acne and weight gain complaints were correlated with tension, depression, and aggression. Studies have shown a positive correlation between body image dissatisfaction and symptoms of anxiety and depression in women (Nilsen et al., 2007). Therefore, factors associated with body image development may also contribute to the observed psychological outcomes.

Conversely, higher estradiol levels were associated with lower aggression scores. The fact that serotonergic transmission in limbic areas and emotional functions is potentiated by estrogen strongly suggests a role of the latter in mood and emotional states in women (Wharton et al., 2012). Moreover, through its effects on PFC and limbic regions (such as the nucleus accumbens), estrogen influences emotional and motivational behaviors, for example, by decreasing impulsive behaviors (Nicola, 2007; Smith et al., 2014). This suggests a potential protective role of estrogen in modulating aggressive behavior (Del Río et al., 2018).

In summary, our findings suggest that hormonal imbalances in adolescents with OD are associated with personal growth and psychological wellbeing domains. Among the limitations of the present study is its cross-sectional nature. Since adolescence is a period of development, longitudinal studies that follow the dimensions studied over time are necessary. Further research is needed to establish causal relationships between the variables considered. In this sense, other aspects, such as early life experiences, previous hormonal contraceptive use, and familiar history of hormonal or psychological conditions, should also be considered. This work could allow a greater understanding of the mechanisms underlying hormonal effects on CNS development, highlighting the importance of considering endocrinological functioning when assessing personal growth in adolescents.

## Data Availability

Due to legal obligations regarding to patient confidentiality and participant privacy, data will be only available if it is requested by qualified researchers and depending on the third party approval. For inquiries about dataset please contact the corresponding author.
